# Inhibition of GSDMD-mediated pyroptosis triggered by *Trichinella spiralis* intervention contributes to the alleviation of DSS-induced ulcerative colitis in mice

**DOI:** 10.1186/s13071-023-05857-3

**Published:** 2023-08-14

**Authors:** Zhen-Rong Ma, Zhuo-Lin Li, Ni Zhang, Bin Lu, Xuan-Wu Li, Ye-Hong Huang, Dibo Nouhoum, Xian-Shu Liu, Ke-Chun Xiao, Li-Ting Cai, Shao-Rui Xu, Xue-Xian O. Yang, Shuai-Qin Huang, Xiang Wu

**Affiliations:** 1 Department of Medical Parasitology, Xiangya School of Basic Medicine, Central South University, Changsha, 410013 Hunan China; 2 Department of Molecular & Genetic and Microbiology, University of New Mexico School of Medicine, Albuquerque, NM 87131 USA

**Keywords:** Trichinella spiralis, Protective effect, Ulcerative colitis, Inhibition, GSDMD-mediated pyroptosis

## Abstract

**Background:**

Inflammatory bowel disease (IBD), including Crohn’s disease (CD) and ulcerative colitis (UC), is increasing worldwide. Although there is currently no completely curative treatment, helminthic therapy shows certain therapeutic potential for UC. Many studies have found that *Trichinella spiralis* (*T.s*) has a protective effect on UC, but the specific mechanism is still unclear.

**Methods:**

Balb/c mice drank dextran sulfate sodium (DSS) to induce acute colitis and then were treated with *T.s.* In vitro experiments, the LPS combination with ATP was used to induce the pyroptosis model, followed by intervention with crude protein from *T.s* (*T.s* cp). Additionally, the pyroptosis agonist of NSC or the pyroptosis inhibitor vx-765 was added to intervene to explore the role of pyroptosis in DSS-induced acute colitis. The degree of pyroptosis was evaluated by western blot, qPCR and IHC, etc., in vivo and in vitro.

**Results:**

*T.s* intervention significantly inhibited NLRP3 inflammasome activation and GSDMD-mediated pyroptosis by downregulating the expression of pyroptosis-related signatures in vitro (cellular inflammatory model) and in vivo (DSS-induced UC mice model). Furthermore, blockade of GSDMD-mediated pyroptosis by the caspase-1 inhibitor vx-765 has a similar therapeutic effect on DSS-induced UC mice with *T.s* intervention, thus indicating that *T.s* intervention alleviated DSS-induced UC in mice by inhibiting GSDMD-mediated pyroptosis.

**Conclusion:**

This study showed that *T.s* could alleviate the pathological severity UC via GSDMD-mediated pyroptosis, and it provides new insight into the mechanistic study and application of helminths in treating colitis.

**Graphical Abstract:**

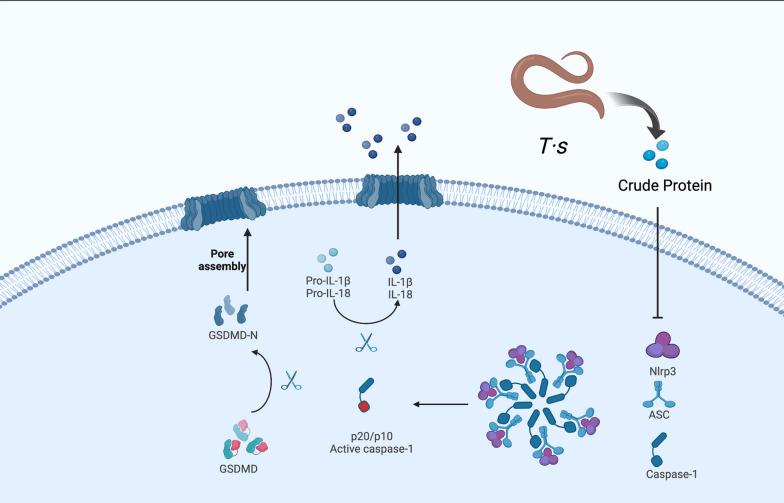

**Supplementary Information:**

The online version contains supplementary material available at 10.1186/s13071-023-05857-3.

## Background

Inflammatory bowel diseases (IBDs), including Crohn’s disease (CD) and ulcerative colitis (UC), are chronic and complex disorders characterized by uncontrolled pathogenic intestinal inflammation and intestinal tissue injury [1]. UC is characterized by long-lasting inflammation, originating in the rectum, and the inflammation is restricted to the mucosal layer of the intestine, resulting in ulceration and bloody stools [2]. The prevalence of UC is increasing globally and occurs widely in people of all ages, and long-term UC patients have an increased risk of developing colorectal cancer [3].

As a kind of autonomic programmed cell death, pyroptosis is closely related to UC [4]. Pyroptosis depends on NLRP3 inflammasome and activated caspase-1 [5]. As innate receptors of the intestinal mucosal immune system, nucleotide-binding oligomerization domain protein-like receptors of NLRP3 assemble into inflammasome complexes with apoptosis-associated speck-like protein (ASC) and proinflammatory caspase-1. On the one hand, activated caspase-1 can cleave inactivated precursors of interleukin-1β (IL-1β) and interleukin-18 (IL-18) into mature IL-1β and IL-18. On the other hand, activated caspase-1 also cleaves gasdermin D (GSDMD) into gasdermin-D N-terminal domain (GSDMD-N) and forms pores in the cell membrane. Thus, the pro-inflammatory cytokines IL-1β and IL-18 are released outside the cell through these pores and cause inflammation. Clinical studies have shown that the NLRP3 protein expression and proinflammatory cytokines of IL-1β were increased in the colon mucosa of most IBD patients [6], and the level of IL-1β in the colon mucosa was positively correlated with disease severity [7]. Most studies have shown that pyroptosis levels were significantly elevated in DSS-induced UC mice [8–14]; therefore, inhibition of pyroptosis may have a therapeutic potential for UC.

There have been several studies regarding the exploitation of helminths to develop novel therapies for the treatment of autoimmune inflammatory diseases including IBD and allergic diseases [15, 16]. *Trichinella spiralis* (*T.s*) and its derived proteins have been investigated for the treatment of many hypersensitivity disorders [17, 18]. Normally, *Trichinella* infection can be caused by ingestion of infected meat [19], and *Trichinella* muscle larvae (ML) are released by digestion of gastric juice and develop into intestinal infectious larvae (IIL) in the intestine. Subsequently, IIL invades the small intestinal epithelium, where they undergo four metamorphoses and develop into adult worms (AW), which mate and produce neonatal larvae (NBL). NBL travel from the intestine to the striated muscle through blood and lymph, eventually developing into L1 stage larvae in muscle cells [20–22]. A number of helminth and helminth-derived products have been shown to have therapeutic effects on UC [16, 23–26]. In addition, helminthic derivatives TSO (*Trichuris suis* ova) and P28 glutathione-*S*-transferase (P28GST) have been shown to have a remission effect on colitis in clinical trials [27, 28]. In recent years, studies on the therapeutic mechanism of worms or their derivatives in UC suggest that worms or their derivatives can relieve inflammation in colitis by regulating the Th1/Th2/Th17 immune response by downregulating proinflammatory cytokines IFN-γ, IL-6 and IL-17 and upregulating anti-inflammatory cytokines IL-4, IL-10 and TGF-β [29–31]. Recent studies have found that the therapeutic effect of *T.s* also correlated with the polarization of M2 macrophages [32], suggesting that the treatment mechanism of worm against colitis may be multifactorial. However, additional mechanisms by which *T.s* improves colitis have not been reported.

Given that *T.s* has a protective effect against DSS-induced UC in mice and has potent anti-inflammatory activity, the present study aimed to examine whether therapeutic effect of *T.s* on colitis was related to the pyroptosis pathway. Our data provided new insights into the mechanisms for the therapeutic application of helminths in the treatment of colitis.

## Methods

### Animals and grouping

SPF Balb/c mice weighing 18–22 g were purchased from the Experimental Animal Center at the School of Basic Medicine of Central South University and housed in a pathogen-free environment in the animal center. All experimental procedures were approved by the Committee of Animal Ethics of Central South University.

### Acquisition of *T. spiralis* crude protein

*Trichinella spiralis* (strain ISS 533) was maintained in KM mice. Muscle larvae were collected from infected mouse muscle by digestion of artificial gastric juice [33, 34]; sedimentation with sterile PBS was repeated several times, and the samples were resuspended in sterile PBS containing 1% penicillin–streptomycin. Then, low-temperature grinding was carried out with a grinder, and the supernatant after centrifugation was *T. spiralis* crude protein (*T.s* cp).

### Establishment of DSS-induced colitis model and treatment

The study was divided into two stages: DSS-induced stage and treatment stage. Mice were assigned randomly to control (*n* = 5) and DSS-induced groups (*n* = 16). Colitis was induced in the DSS group by the administration of 3.5% DSS (molecular weight: 36–50 kDa; MP Biomedicals, Irvine, CA, USA) for 7 days. Then, the DSS group mice were assigned to the UC model and treatment groups, with eight animals per group. The treatment groups were 300 administered *T.s* larvae. At the end of the experiment, the mice were fasted for 24 h and killed, and the colons were collected. Colon lengths were recorded, and colon specimens were frozen in liquid nitrogen or immediately fixed in 4% paraformaldehyde for further analysis.

### Analysis of myeloperoxidase activity

Myeloperoxidase (MPO) reflects the function and activity of neutrophils in tissues. MPO levels were measured using an MPO ELISA kit (Nanjing Jiancheng Technology Co., Ltd., Nanjing, China) according to the manufacturer’s instructions.

### Evaluation of colitis severity

The severity of colitis was evaluated based on body weight, colon length, and macroscopic and microscopic observations of the stool and colon. The disease activity index (DAI) score was evaluated according to the method reported in previous studies [35]. The DAI assessment was determined by the scoring criteria described in Additional file 2: Table S1. Colon sections were prepared and stained with hematoxylin and eosin (H&E) by Wuhan Saiweier Biological Technology Co., Ltd. (Wuhan, China), and the histology score was evaluated by two parameters of epithelial damage and infiltration extent according to the previous study [29]. All evaluations were performed blind.

### RNA extraction and quantitative real-time PCR (qRT-PCR) assay

Total RNA from colonic tissues and cells was isolated with Trizol (TransGen Biotech, Beijing, China) and reverse transcribed using the reverse transcription kit (TransGen Biotech, China). qPCR was performed using SYBR Green reagent (TransGen Biotech). Primer sequences are listed in Additional file 3: Table S2, and GAPDH was used as a reference. The quantitative PCR conditions were: 94 °C for 30 s, 40 cycles of 94 °C for 5 s, 60 °C for 15 s and 72 °C for 10 s. The relative expression level of each gene was calculated by the 2^−ΔΔCt^ method [36], and the experiment was repeated three times.

### Western blot assay

Colon tissues and cells were homogenized in RIPA buffer (Beyotime Biotechnology, Shanghai, China) with a phosphatase inhibitor cocktail (Beyotime). Protein was separated by SDS-PAGE (Beyotime) and transferred to PVDF membranes (Millipore) as previously reported [37]. Membranes were blocked with 5% skim milk solution for 2 h and incubated with the corresponding primary antibody at 4 °C overnight. Then, the membranes were incubated with secondary antibody for 2 h at room temperature. The protein bands were visualized using ECL reagents. Antibodies used are listed in Additional file 4: Table S3. The relative band intensity was measured using Image J software and further used for statistical analysis (Additional files 2, 3).

### Interventions with pyroptosis agonists and inhibitors

The pyroptosis agonist nigericin (NSC 292567) and inhibitor belnacasan (vx-765) were purchased from Selleck Chemicals, LLC (Houston, TX, USA). DSS was induced in the experimental group by the administration of 3.5% DSS (molecular weight: 36–50 kDa; MP Biomedicals) for 7 days (*n* = 5), and the treatment group mice were given a dose of 50 mg/kg VX-765 (pyroptosis inhibitor) (*n* = 5), 50 mg/kg NSC (pyroptosis agonist) (*n* = 5) and DMSO control (*n* = 5) for 5 days. At the end of the experiment, after fasting for 24 h, the mice were killed and the colons collected. Colon lengths were recorded, and colon specimens were frozen in liquid nitrogen or immediately fixed in 4% paraformaldehyde for further analysis.

### Cell culture

RAW264.7 and MODE-K cells were purchased from Whelab (Shanghai Yingwan Biological, Shanghai, China) and cultured in 5% CO_2_, 37 °C.

### Induction of cell pyroptosis in vitro

Cells were seeded in six-well plates. After 24 h of culture, the cells were stimulated with 1 µg/ml LPS for 6 h and co-cultured with 5 mM ATP (or 40 µM NSC) for another 2 h before collection. *T.s* crude protein (4 μg/ml) was added and then incubated for 30 min before adding ATP (or NSC).

### Statistical analysis

GraphPad Prism 8 was used for statistical analyses. All results are presented as mean ± SD, and the differences between groups were analyzed using one-way ANOVA analysis. Statistics symbols used are as follows: *P < 0.05, **P < 0.01, ***P < 0.001.

## Results

### *T.s* intervention suppresses GSDMD-mediated pyroptosis in DSS-induced colitis mice

**Fig. 1:**
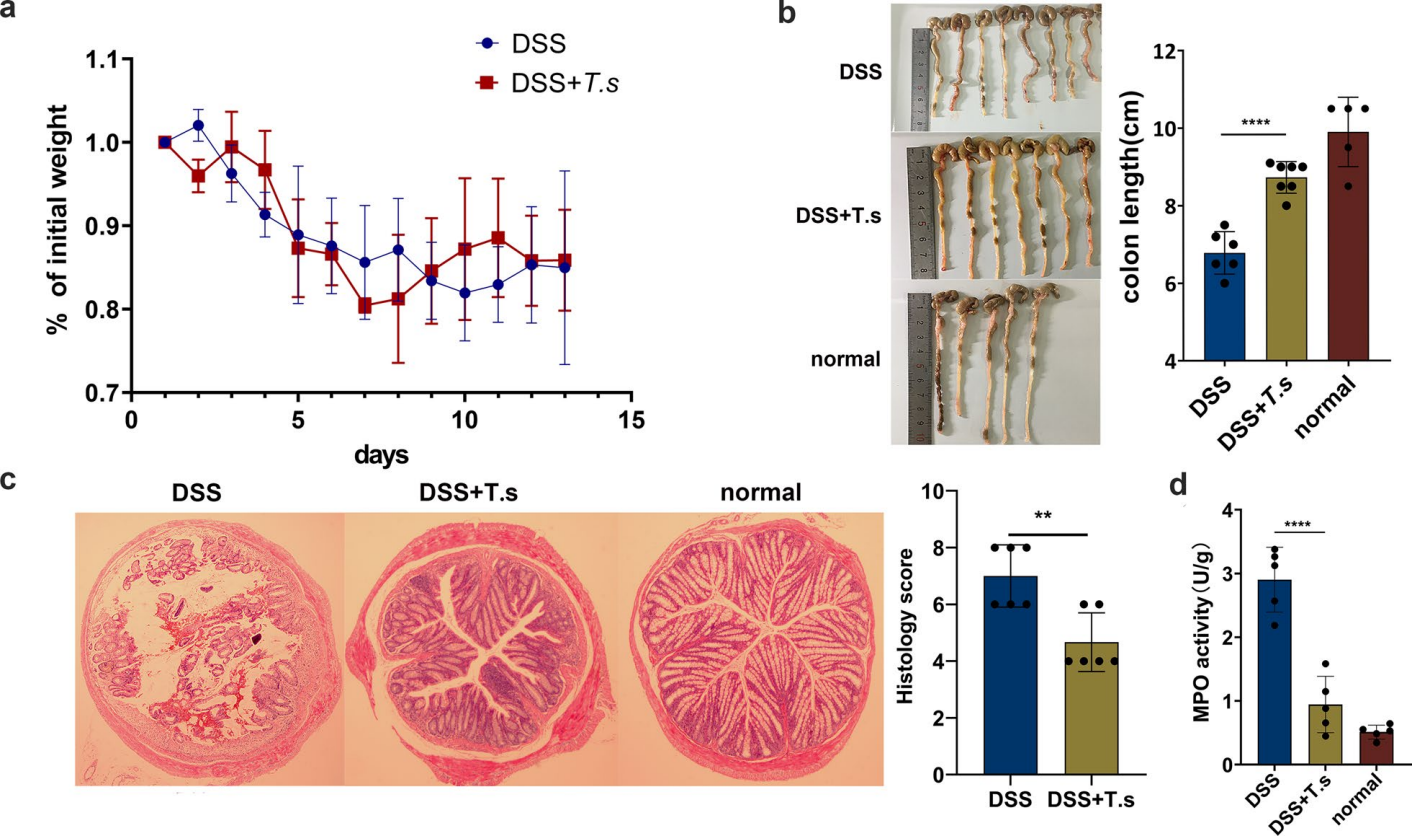
*T.s* intervention attenuated clinical symptoms in DSS-induced UC mice. **a** The body weights were recorded and analyzed. **b** Colonic length was measured and recorded. **c** Histopathological changes in colonic samples were examined by H&E staining (40 ×). Histology scores (right) in each group. **d** MPO activity detection. All the results are presented as mean ± SD, and statistics symbols used are: *P < 0.05, **P < 0.01, ***P < 0.005, ****P < 0.001

Compared with the control mice, the body weights of the mice treated with DSS were continuously decreased until 1 or 2 days after stopping DSS induction. After *T.s* administration, the body weights of the mice increased after aborting DSS induction (Fig. 1a). Colon length in the *T.s* groups was longer than that in the DSS group (Fig. 1b). During the experiment, symptoms of UC, such as diarrhea and hemafecia, were less severe in the *T.s* groups. Colon damage in the *T.s* groups was much less than that in the DSS group. In addition, the DAI score and histological damage were decreased in colitis mice after treatment with *T.s* (Fig. 1c). MPO levels were measured to assess the effects of *T.s* on colonic epithelial damage and neutrophil infiltration. The results showed that *T.s* treatment significantly reduced MPO activity in DSS-induced colitis mice (Fig. 1d). These data indicated that *T.s* intervention could relieve the clinical symptoms of DSS-induced colitis mice.

In addition, compared with the control group, the levels of the inflammatory cytokines TNF-α and IL-6 were significantly increased in the DSS group. *T.s* treatment significantly reduced the levels of the proinflammatory cytokines and increased anti-inflammatory factors IL-4, IL-13, IL-10 and TGF-β (Additional file 1: Figure S1). Furthermore, our results showed that IL-22 and iNOS levels were significantly elevated in the DSS group (Additional file 1: Figure S1).

**Fig. 2:**
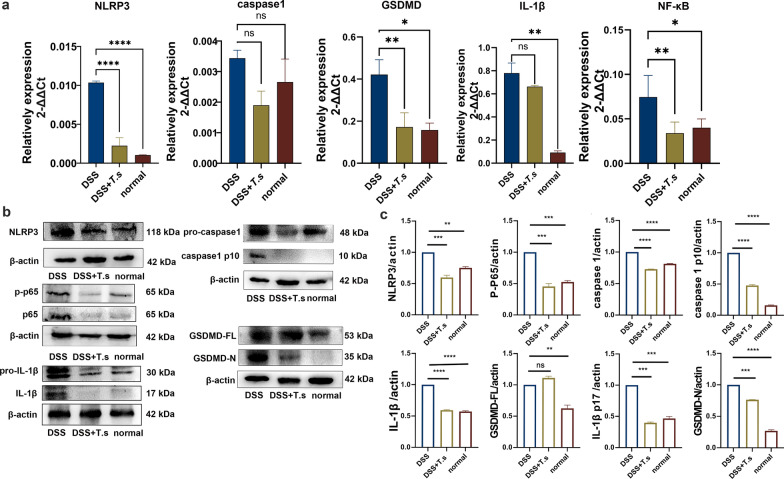
*T.s* intervention downregulates the expression levels of pyroptosis-associated molecules in DSS-induced ulcerative colitis mice. **a** qPCR detection of the relative expression levels of NLRP3, caspase-1, GSDMD, IL-1β and NF-κB. **b**–**c** Total proteins were extracted; about 40 μg protein was added to each lane, and specific antibodies were used to detect the relative expression levels of NLRP3, caspase-1, caspase-1 p10, GSDMD, GSDMD-N, IL-1β and p17 and phosphorylated NF-κB (p65). All the results are presented as mean ± SD, and statistics symbols used are: *P < 0.05, **P < 0.01, ***P < 0.005, ****P < 0.001

**Fig. 3:**
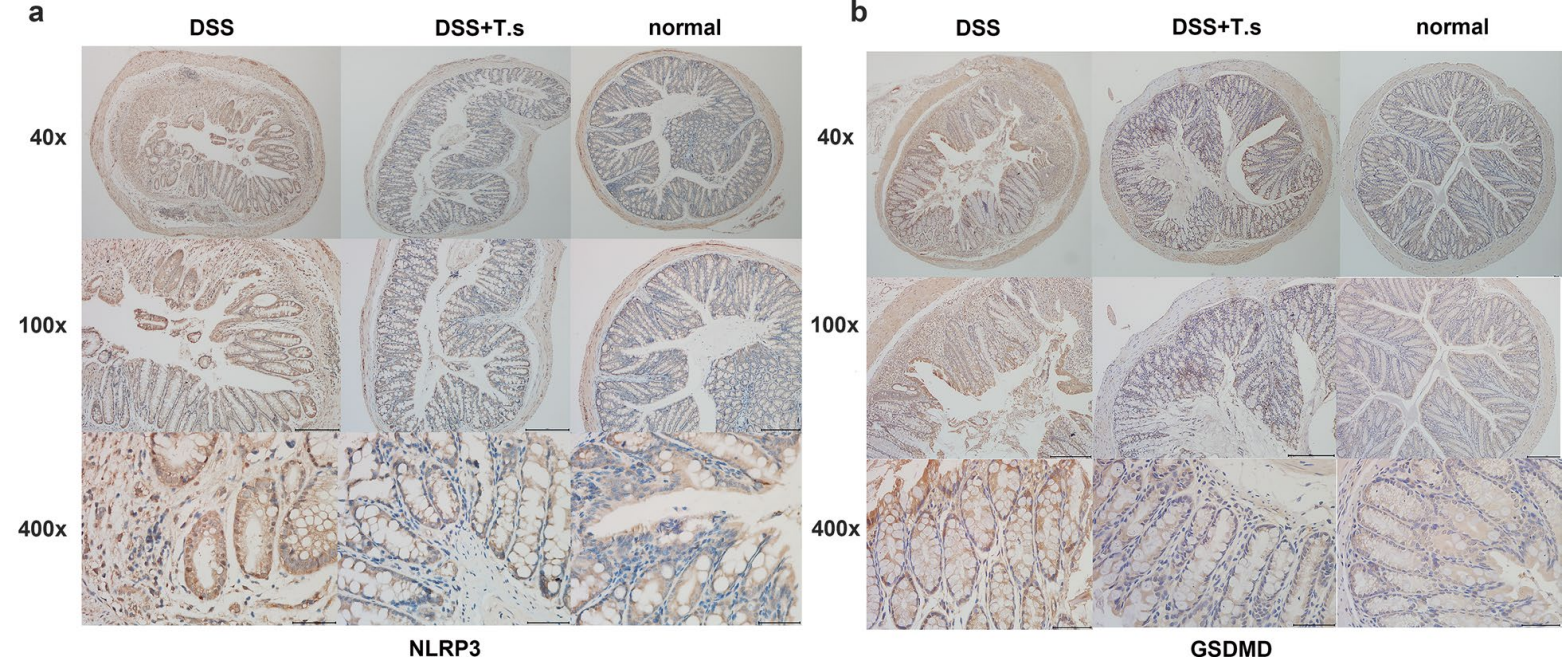
Immunohistochemical detection of NLRP3 **a** and GSDMD **b** in mouse colon tissues in DSS-induced ulcerative colitis mice

Pyroptosis has recently been verified as a critical cell death pathway in colitis, and caspase-1/GSDMD-mediated pyroptosis is an inflammatory cell death process that has become a novel target for UC treatment [38]. In this study, we attempted to investigate the effect of *T.s* or *T.s* crude proteins on the pyroptosis pathway. The results showed that the mRNA expression levels of NLRP3, GSDMD and NF-κB were highly expressed in the colon tissues of DSS-induced colitis and significantly decreased in the *T.s* intervention groups. However, the relative mRNA expression of caspase-1 and IL-1β was not statistically different between the DSS and *T.s* treatment group (Fig. 2a). Moreover, we also found that the corresponding protein levels showed the same trend as mRNA, with decreased protein levels of NLRP3, caspase-1, caspase-1 p10, GSDMD-N, IL-1β and IL-1β p17 in the *T.s* groups. In addition, the phosphorylation level of p65 of the mouse colons in the *T.s* group was significantly decreased as well (Fig. 2b–c). Next, the immunohistochemical assay was conducted to validate the expression levels of pyroptosis indicators NLRP3 and GSDMD in the colon tissue, and the results showed that the expression levels in the *T.s* treatment group were significantly lower than those in the DSS group (Fig. 3). These results indicated that *T.s* intervention could inhibit NLRP3 inflammasome activation and GSDMD-mediated pyroptosis in DSS-induced colitis mice.

### *T.s* crude proteins treatment inhibits GSDMD-mediated pyroptosis in macrophage inflammatory model of RAW264.7 cells

**Fig. 4:**
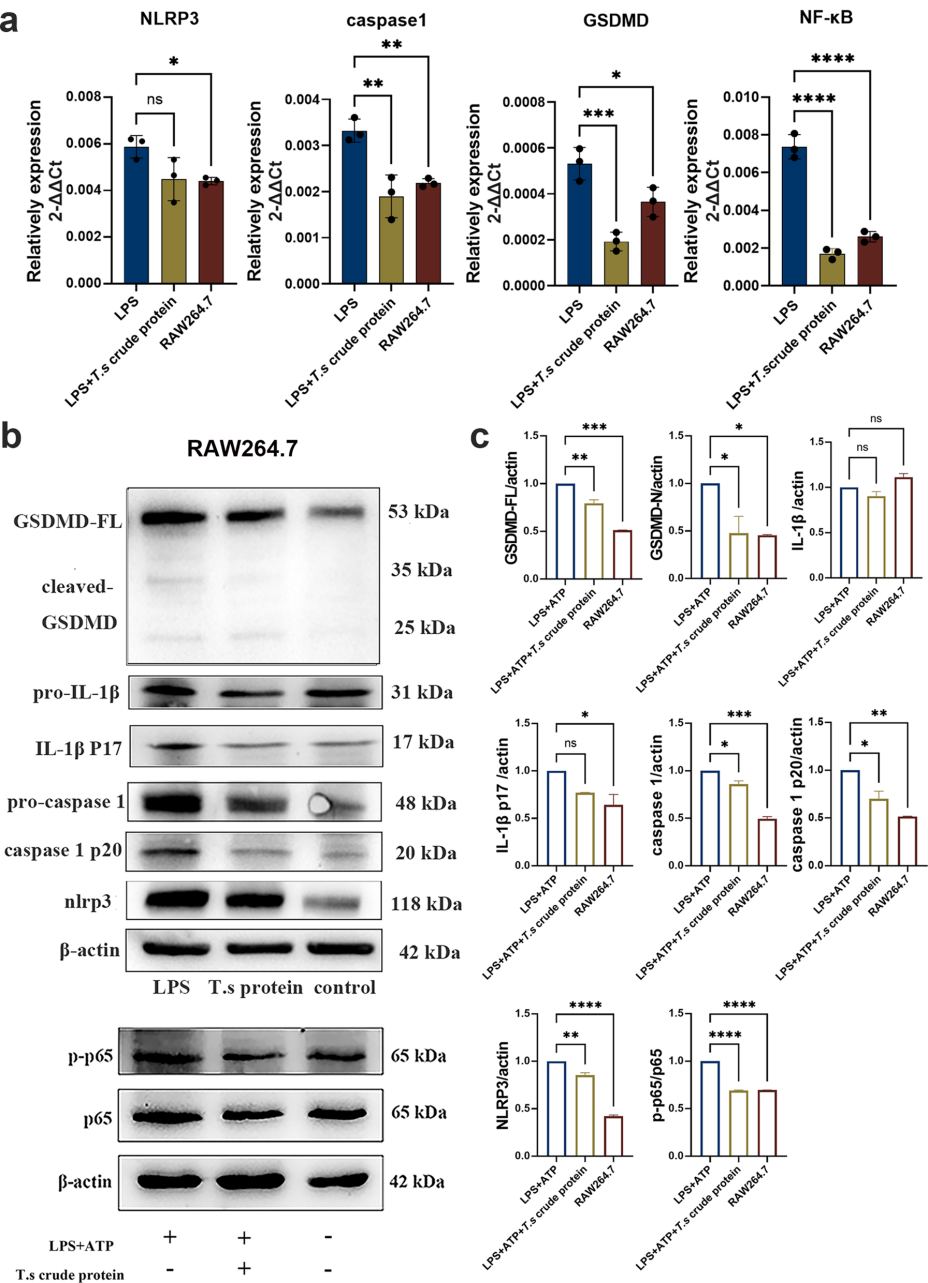
*T.s* crude protein treatment downregulated the expression of pyroptosis-associated molecules in RAW264.7 cells. **a** *T.s* crude protein treatment downregulated mRNA expression levels of pyroptosis-associated molecules of NLRP3, caspase-1, GSDMD and NF-κB induced by LPS and ATP. **b**–**c** Western blot was used to detect the relative expression level of GSDMD-FL, GSDMD-N, caspase-1, caspase-1 p20, IL-1β, IL-1β p17 and phosphorylated NF-κB (p65). All the results are presented as mean ± SD, and statistics symbols used are: *P < 0.05, **P < 0.01 ***P < 0.005, ****P < 0.001

Clinical studies and animal model experiments have reported that NLRP3 inflammasome activation and GSDMD-mediated pyroptosis are closely related to IBD. A macrophage inflammatory model was used to verify the effect of *T.s* intervention on the pyroptosis-related pathway. The results showed that after treatment with *T.s* crude proteins, the mRNA expression levels of GSDMD, NLRP3, caspase-1 and NF-κB (Fig. 4a) were significantly decreased in RAW264.7 cells induced by LPS and ATP. Furthermore, the protein levels of GSDMD-FL, GSDMD-N, NLRP3, caspase-1 p20, IL-1β, IL-1β p17 and phosphorylated NF-κB (p65) were also significantly decreased in the *T.s* crude protein groups (Fig. 4b–c). These data indicated that *T.s* crude protein treatment could suppress NLRP3 inflammasome activation and GSDMD-mediated pyroptosis in a macrophage inflammatory model of RAW264.7 cells.

### *T.s* crude proteins treatment inhibits GSDMD-mediated pyroptosis in an intestinal epithelial cellular inflammatory model of MODE-K cells

**Fig. 5:**
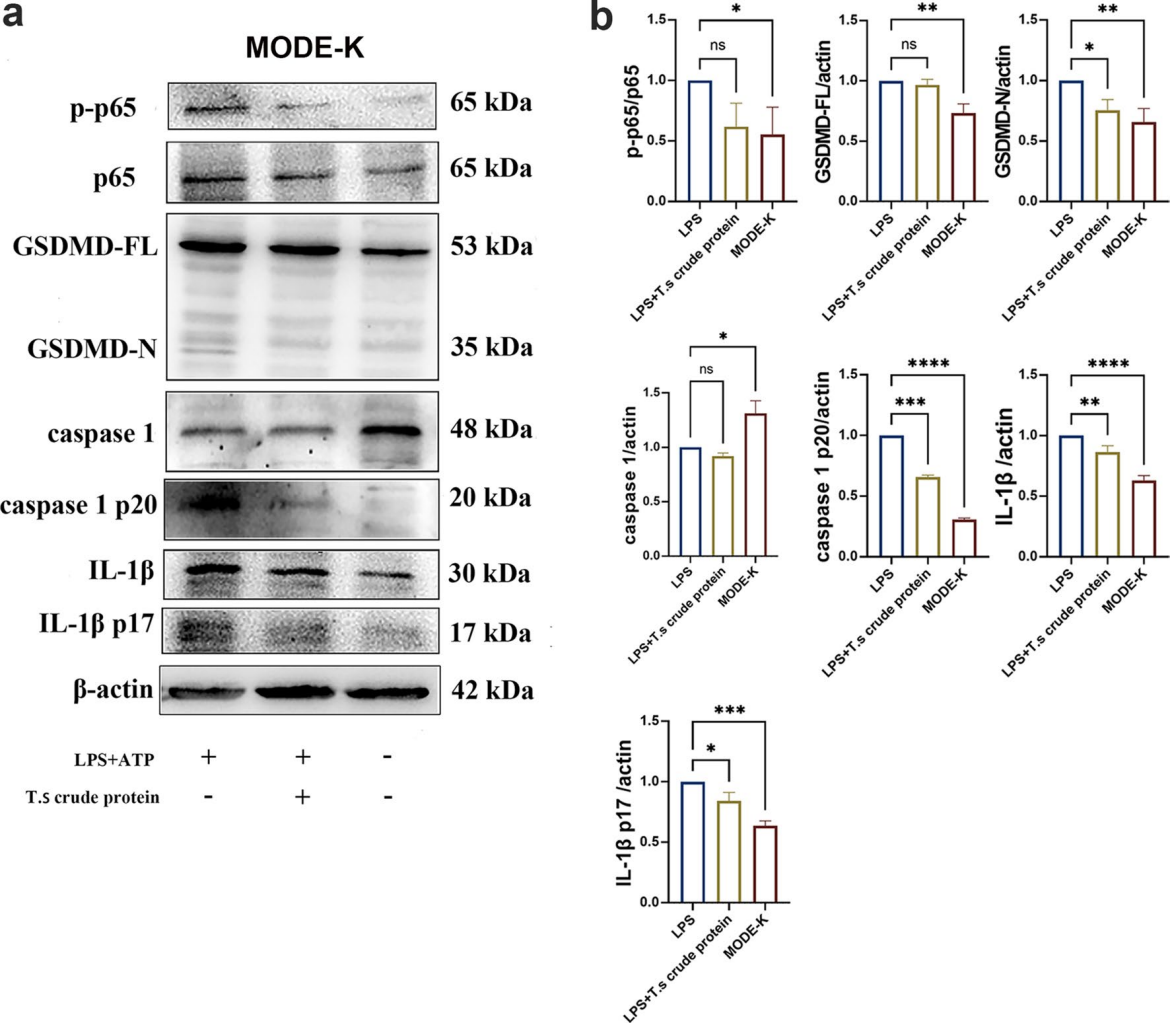
*T.s* crude protein treatment downregulated the expression of pyroptosis-associated molecules in MODE-K cells. **a** Western blot detected the relative expression levels of GSDMD-FL, GSDMD-N, caspase-1, caspase-1 p20, IL-1β, IL-1β p17 and phosphorylated NF-κB (p65). **b** Grayscale scan values for the results obtained from western blot analysis. All results are presented as mean ± SD, and statistics symbols used are: *P < 0.05, **P < 0.01, ***P < 0.005, ****P < 0.001

It has been reported that GSDMD are produced by gut mucosa intestinal epithelial cells (IECs) in colitis model mice, and a deficiency of GSDMD effectively reduced the severity of dextran sodium sulfate (DSS)-induced colitis [39, 40]. Therefore, the mechanisms regulating IEC survival and death are critical for immune homeostasis and pathogenesis in intestinal inflammatory diseases. A recent study showed that LPS also induced IL-1β and GSDMD expression in colonic IECs [41]. Our results showed that the protein levels of GSDMD-N, caspase-1 p20, IL-1β and IL-1β p17 were decreased in the *T.s* crude protein groups in the intestinal epithelial cell line of MODE-K cell-induced pyroptosis model. Phosphorylation levels of NF-κB(p65) protein in the *T.s* crude protein group were also significantly decreased (Fig. 5). The results demonstrated that *T.s* crude protein treatment could inhibit GSDMD-mediated pyroptosis by downregulating the expression of pyroptosis-related molecules in an intestinal epithelial cellular inflammatory model of MODE-K cells.

### Blockade of caspase-1/GSDMD-mediated pyroptosis ameliorates DSS-induced colitis in mice

**Fig. 6:**
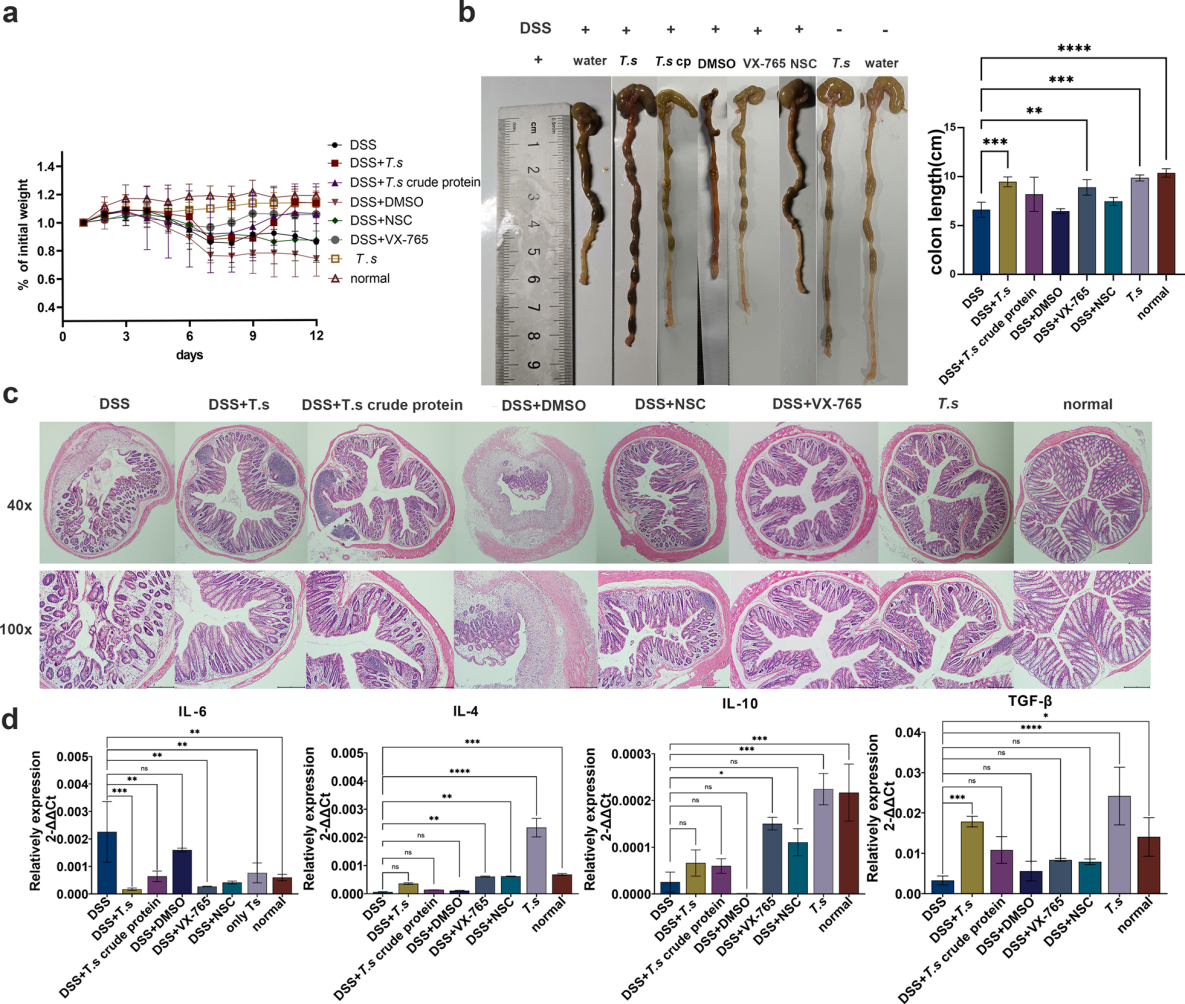
Pyroptosis inhibitor vx-765 relieved DSS-induced colitis. **a** The body weight changes in DSS-induced colitis after pyroptosis inhibitor belnacasan (vx-765) and pyroptosis agonist nigericin (NSC) interventions. **b** Colonic length in all the experimental groups were measured and recorded. **c** Histopathological changes in colonic samples were examined by H&E staining and were measured by evaluating its therapeutic effect. **d** Cytokines of IL-6, TGF-β, IL-4 and IL-10 were measured. All the results are presented as mean ± SD, and statistics symbols used are: *P < 0.05, **P < 0.01, ***P < 0.005, ****P < 0.001

Caspase-1 activation could mediate GSDMD cleavage and IL-1β secretion [5]. To further understand the role of pyroptosis in DSS-induced colitis, the inhibitor belnacasan (vx-765) and pyroptosis agonist nigericin (NSC) were used to intervene the experimental mice. This showed that pyroptosis inhibitor vx-765 intervention improved the clinical symptoms of DSS-induced colitis mice with reduced weight loss (Fig. 6a), shortened colon length (Fig. 6b) and decreased severity of pathological sections (Fig. 6c). Similar to the therapeutic effect of *T.s* treatment, after vx-765 intervention, pro-inflammatory cytokine IL-6 in DSS-induced colitis mice was significantly reduced, while anti-inflammatory cytokines such as IL-4, IL-10 and TGF-β were significantly increased (Fig. 6d), indicating blockade of caspase-1-mediated pyroptosis could balance the immune disorder of DSS-induced colitis in mice.

**Fig. 7:**
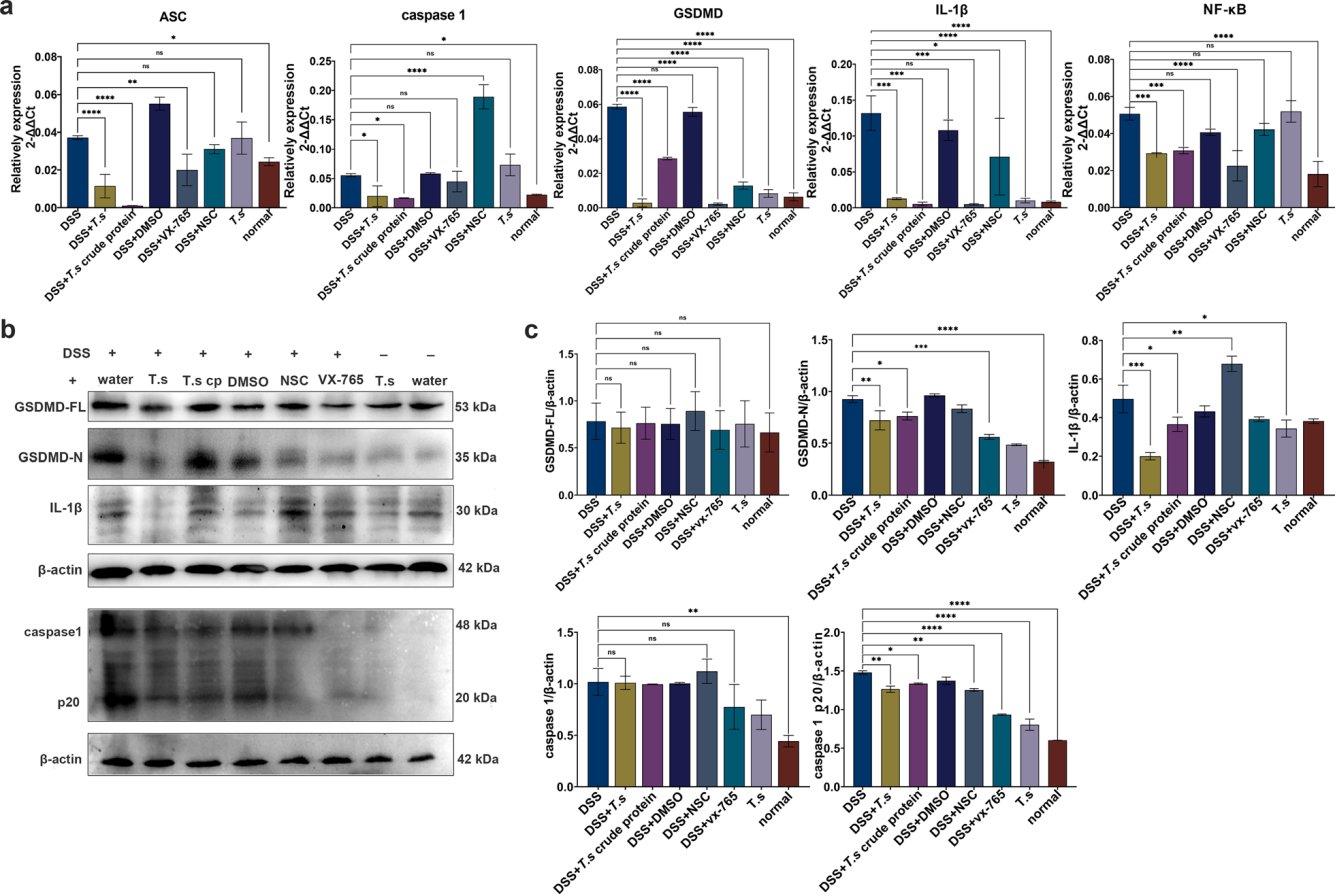
*T.s* or vx-765 intervention relieved DSS-induced pyroptosis in colitis mice. **a** qPCR detection of the relative expression levels of pyroptosis-related molecules ASC, caspase-1, IL-1β, GSDMD and NF-κB. **b** Western blot was used to determine the relative expression levels of GSDMD, GSDMD-N, IL-1β, caspase-1 and caspase-1 p20. **c** Grayscale scan values for the results obtained from western blot analysis. All the results are presented as mean ± SD, and statistics symbols used are: *P < 0.05, **P < 0.01, ***P < 0.005, ****P < 0.001

Further study showed that mRNA expression levels of ASC, caspase-1, GSDMD, IL-1β and NF-κB were decreased significantly in both the *T.s* crude protein and the vx-765 intervention groups (Fig. 7a). The protein levels of GSDMD-N, caspase-1 p20 and IL-1β were decreased in the *T.s* group, *T.s* crude protein group and vx-765 intervention group (Fig. 7b–c). This illustrated that both blockade of caspase-1-mediated pyroptosis and *T.s* intervention could ameliorate DSS-induced colitis in mice.

## Discussion

Increasing evidence has suggested that helminths and their secreted products have therapeutic potential in the treatment of inflammatory diseases, including IBD [42]. Growing numbers of epidemiological investigations in different regions of the world have found an inverse relationship between the prevalence of autoimmune diseases like IBD and parasitic infections [43]. Helminths indeed have the ability to protect against or alleviate numerous inflammatory conditions, such as IBD, allergic airway inflammation, type I diabetes and autoimmune encephalomyelitis [44]. Therefore, helminth therapy has been an attractive autoimmune therapy approach.

*T.s* infection can activate the Th2 immune response, and cytokines of the Th2 subpopulation can induce unique immunological features such as eosinophilia, mastocytosis and IgE hypergammaglobulinemia [45]. A Th2-biased immune response is caused by *T.s* infection, characterized by Th2-associated cytokines, typically including IL-4, IL-5 and IL-13. In addition, *T.s* can regulate Th1 and Th2 responses by inducing regulatory T cells (Treg) or anti-inflammatory cytokines IL-10 and TGF-β [46]. Several studies have focused on the anti-inflammatory property of *T.s* or their derived products in an IBD model and revealed that the protective mechanism was related to Th2 cells by balancing Th1/Th2/Th17 population [47, 48]. In this study, the levels of pro-inflammatory cytokines, such as IL-6, were increased in the UC mouse intestinal tissues, and anti-inflammatory factors, such as IL-4, IL-10 and TGF-β, were obviously elevated in the *T.s* treatment group, consistent with the findings of other investigations [35, 49–51]. In general, IL-22 plays a protective role in IBD, and although IL-22 levels were elevated in DSS-induced UC mice, it may have been accompanied by regulation of the natural antagonist IL-22BP, thereby hindering the protective effect of IL-22. In addition, iNOS was significantly elevated in the DSS group, which may be related with the polarization of macrophages toward the M1 type in colitis. These results demonstrated that *T.s* intervention could regulate immune balance in DSS-induced colitis mice.

It is known that pyroptosis plays a crucial role in inflammation occurrence and the development of UC diseases. The over-activation of the NLRP3/caspase-1 pathway is a key step of pyroptosis [52]. NLRP3 inflammasome activation requires two distinct signals; the first signal is NF-κB-mediated NLRP3, where pro-IL-1β and IL-18 expression is regulated by inflammatory stimuli such as TLR4 agonists. The second signal is the assembly of NLRP3 inflammasome, caspase-1 activation and IL-1β and IL-18 secretion [53, 54]. In our study, the transcription level of NF-κB (p65) was significantly increased in the UC mouse intestinal tissue and in vitro, consistent with the results reported by Chen et al. [55], revealing that NF-κB pathway plays a profound regulatory role in the pathogenesis of colitis and NLRP3 activation could rely on NF-κB pathway.

It has been demonstrated that NLRP3 inflammasome activation leads to GSDMD cleavage activation and IL-1β release in response to caspase1 activation in a variety of cell types, including neutrophils, macrophages, dendritic cells and human monocytes [56, 57]. NLRP3 inflammasome activation leads to autocrine IL-1β signaling to propagate and amplify the inflammatory response and inflammatory phenotype and increase the persistent IL-1β in intestinal lesions and mucosal cells from IBD patients [58]. In the present study, the protein levels of active caspase-1 (caspase p20), NLRP3, IL-1β and IL-1β p17 were significantly increased in the UC mouse intestinal tissue and MODE-K, RAW264.7 cells treated with LPS and ATP in vitro, and *T.s* or *T.s* crude protein intervention significantly suppressed accumulation of the NLRP3 inflammasome and activation of caspase-1 pathway. However, there is no statistical difference in the mRNA level of caspase-1, suggesting that *T.s* intervention did not have an effect on the transcription of caspase-1. Additionally, the pyroptosis effector of N-terminus of GSDMD (GSDMD-N) was elevated in a UC mouse model and cell model in vitro and decreased after *T.s* or *T.s* crude protein treatment. Therefore, it was believed that pyroptosis plays an important role in the progression of UC and could be suppressed by *T.s* intervention. In addition, the GSDMD and oxidative stress-related molecule iNOS were significantly elevated in the UC mice, consistent with the previous study [59].

In addition, the pathological severity of DSS-induced UC mice was alleviated by caspase-1 inhibitor vx-765 intervention. In addition, the mRNA and protein levels of key pyroptosis-related molecules including caspase-1, GSDMD and IL-1β of UC mice in the vx-765 treatment group and the *T.s* treatment group were significantly downregulated compared with those in the DSS group. In summary, our data indicated that the protective effect on DSS-induced UC by *T.s* intervention was achieved through the inhibition of GSDMD-mediated pyroptosis.

## Conclusions

This study showed that *T.s* could alleviate the pathological severity UC via GSDMD-mediated pyroptosis and provides new insight into the mechanism study and application of helminths in treating colitis. Our results help to better understand the mechanisms involved in the inverse correlation between parasitic helminth infection and incidence of immune-mediated inflammatory diseases including IBD.

### Supplementary Information

**Additional file 1: Figure S1**. *T.s* treatment balanced the Th1/Th2/Th17 immune response in DSS-induced UC mice. qPCR detection of the relative mRNA expression levels of IL-13, IL-22, IL-10, TGF-β, iNOS, TNF-α, IL-6 and IL-4. All results are presented as mean ± SD, and statistics symbols used are: *P < 0.05, **P < 0.01, ***P<0.005, ****P< 0.001

**Additional file 2: Table S1.** Assessment of the disease activity index (DAI).

**Additional file 3: Table S2.** Primers used in the qRT-PCR experiment.

**Additional file 4: Table S3.** Antibodies used in the western blot experiment.

## Data Availability

The datasets supporting the findings of this article are included within the paper and its Additional file materials.

## Abbreviations

CD: Crohn’s disease
DSS: Dextran Sulfate Sodium Salt
DAI: Disease activity index
GSDMD: Gasdermin-D
H&E: Hematoxylin and eosin
IBD: Inflammatory bowel disease
IL: Interleukin
IFN: Interferon
IECs: Intestinal epithelial cells
LPS: Lipopolysaccharide
MPO: Myeloperoxidase
NLRs: Nucleotide-binding oligomerization domain protein-like receptors
NF-κB: Nuclear factor-kappa B
qRT-PCR: Quantitative real-time PCR
STAT: Signal transducer and activator of transcription
TNF: Tumor necrosis factor
TGF: Transforming growth factor
*T.s*: *Trichinella spiralis*
Th: T help cell
UC: Ulcerative colitis
