# Supplementary figures and images for: Inhibition of GSDMD-mediated pyroptosis triggered by Trichinella spiralis intervention contributes to the alleviation of DSS-induced ulcerative colitis in mice
Source: Parasit Vectors. 2023 Aug 14;16:280. doi: 10.1186/s13071-023-05857-3 (PMC10424392; doi:10.1186/s13071-023-05857-3)

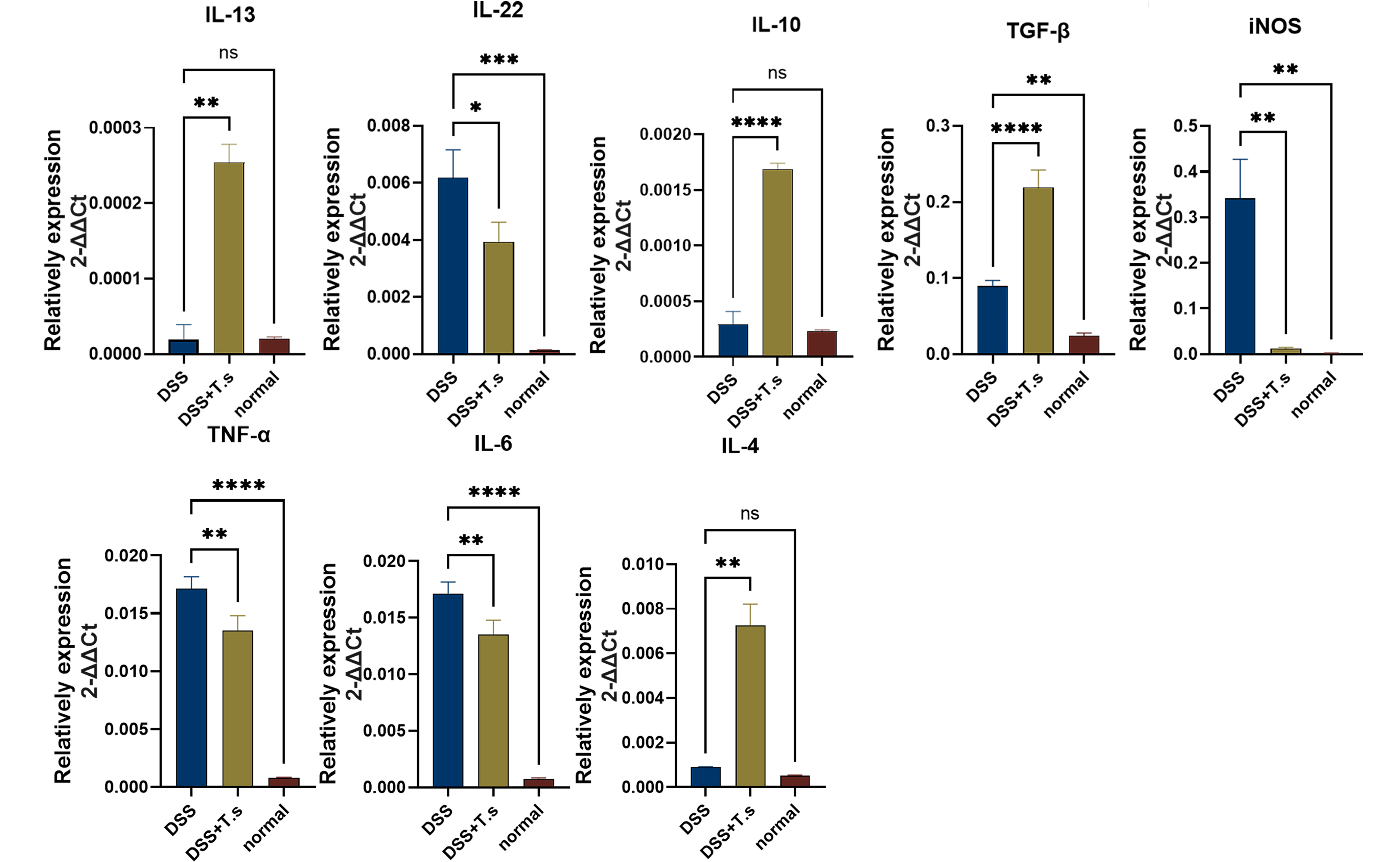

Supplement: Supplementary file 1 — Additional file 1: Figure S1. T.s treatment balanced the Th1/Th2/Th17 immune response in DSS-induced UC mice. qPCR detection of the relative mRNA expression levels of IL-13, IL-22, IL-10, TGF-β, iNOS, TNF-α, IL-6 and IL-4. All results are presented as mean ± SD, and statistics symbols used are: *P < 0.05, **P < 0.01, ***P<0.005, ****P< 0.001 [file 13071_2023_5857_MOESM1_ESM.tif]
