# Supplementary material for: Inhibition of GSDMD-mediated pyroptosis triggered by Trichinella spiralis intervention contributes to the alleviation of DSS-induced ulcerative colitis in mice
Source: Parasit Vectors. 2023 Aug 14;16:280. doi: 10.1186/s13071-023-05857-3 (PMC10424392; doi:10.1186/s13071-023-05857-3)
Supplement: Supplementary file 2 — Additional file 2: Table S1. Assessment of the disease activity index (DAI). [file 13071_2023_5857_MOESM2_ESM.docx]

**Supplementary table 1**

**Assessment of the disease activity index (DAI)**

| **Body weight loss (%)** | **Stool** | **Bleeding** | **score** |
| --- | --- | --- | --- |
| <2% | Normal | No rectal bleeding | 0 |
| 2%-<5% | Softer stool | Weak haemoccult | 1 |
| 5%-<10% | Moderate  diarrhoea | Visual blood in stool | 2 |
| 10%-<15% | Diarrhoea | Fresh rectal bleeding | 3 |
| > 15% | - | - | 4 |

**Supplementary table 2**

**Primer sequences**

| **gene** | **Primer sequence** |
| --- | --- |
| GAPDH | F: TGTTTCCTCGTCCCGTAGA  R: ATCTCCACTTTGCCACTGC |
| IL-10 | \| F: AGCCGGGAAGACAATAACTG \| \| --- \| \| R: CATTTCCGATAAGGCTTGG \| |
| TGF-β | \| F: AACTATTGCTTCAGCTCCACAG \| \| --- \| \| R: AGTTGGCATGGTAGCCCTTG \| |
| IL-1β | \| F: CTCACAAGCAGAGCACAAGC \| \| --- \| \| R: TCCAGCCCATACTTTAGGAAGA \| |
| NF-κB | \| F \| GAGGAAGGCTGTGAACATGAGG \| \| --- \| --- \| \| R \| TTCTGGTGCATTCTGACCTTGC \| |
| NLRP3 | \| F \| AGA TTA CCC GCC CGA GAA AG \| \| --- \| --- \| \| R \| TCC CAG CAA ACC CAT CCA CT \| |
| Arg-1 | \| F \| AACACTCCCCTG ACAACCA \| \| --- \| --- \| \| R \| CATCACCTTGCCAATCCC \| |
| iNOS | \| F \| CAGCTGGGCTGTACAAACCTT \| \| --- \| --- \| \| R \| CATTGGAAGTGAAGCGTTTCG \| |
| IL-22 | \| F: CATGCAGGAGGTGGTACCTT \| \| --- \| \| R: CAGACGCAAGCATTTCTCAG \| |
| TNF-alfa | \| F: CCCTCACACTCAGATCATCTTCT \| \| --- \| \| R: GCTACGACGTGGGCTACAG \| |
| IL-6 | F: TAGTCCTTCCTACCCCAATTTCC  R: TTGGTCCTTAGCCACTCCTTC |
| GSDMD | \| F \| ATCCTGGCATTCCGAGTGG \| \| --- \| --- \| \| R \| CTCTGGCCCACTGCTTTTCT \| |
| pro-caspase-1 | \| F: CACAGCTCTGGAGATGGTGA \| \| --- \| \| R: CTTTCAAGCTTGGGCACTTC \| |
| ASC | \| F: GACAGTACCAGGCAGTTCGT \| \| --- \| \| R: AGTCCTTGCAGGTCAGGTTC \| |

**Supplementary table 3**

**Antibodies**

| **Antibody** | **Company and catalog** |
| --- | --- |
| NLRP3 | Recombinant Anti-NLRP3 antibody  Abcam (ab263899) |
| IL-1β | Recombinant Anti-IL-1 beta antibody  Abcam (ab254360) |
| GSDMD | Recombinant Anti-GSDMD antibody  Abcam (ab219800) |
| GSDMD-N | GSDMDC1 Antibody (64-Y):  santa cruz sc-81868 |
| Caspase1 | Caspase1 (E2Z1C) Rabbit mAb  CST #24232 |
| Caspase1 p20 | caspase-1 p20 Antibody (D-4)  santa cruz sc-398715 |
| NF-κB (p65) | NF-kB p65 Antibody  SAB Catalog No: #48676 |
| p-NF-κB (p-p65) | Phospho-NF-κB p65 (Ser536) (93H1) Rabbit mAb  CST #3033 |
