# Supplementary material for: Inhibition of GSDMD-mediated pyroptosis triggered by Trichinella spiralis intervention contributes to the alleviation of DSS-induced ulcerative colitis in mice
Source: Parasit Vectors. 2023 Aug 14;16:280. doi: 10.1186/s13071-023-05857-3 (PMC10424392; doi:10.1186/s13071-023-05857-3)
Supplement: Supplementary file 3 — Additional file 3: Table S2. Primers used in the qRT-PCR experiment. [file 13071_2023_5857_MOESM3_ESM.docx]

**Supplementary table 2**

**Primer sequences**

| gene | Primer sequence |
| --- | --- |
| GAPDH | F: TGTTTCCTCGTCCCGTAGA  R: ATCTCCACTTTGCCACTGC |
| IL-10 | \| F: AGCCGGGAAGACAATAACTG \| \| --- \| \| R: CATTTCCGATAAGGCTTGG \| |
| TGF-β | \| F: AACTATTGCTTCAGCTCCACAG \| \| --- \| \| R: AGTTGGCATGGTAGCCCTTG \| |
| IL-1β | \| F: CTCACAAGCAGAGCACAAGC \| \| --- \| \| R: TCCAGCCCATACTTTAGGAAGA \| |
| NF-κB | \| F \| GAGGAAGGCTGTGAACATGAGG \| \| --- \| --- \| \| R \| TTCTGGTGCATTCTGACCTTGC \| |
| NLRP3 | \| F \| AGA TTA CCC GCC CGA GAA AG \| \| --- \| --- \| \| R \| TCC CAG CAA ACC CAT CCA CT \| |
| Arg-1 | \| F \| AACACTCCCCTG ACAACCA \| \| --- \| --- \| \| R \| CATCACCTTGCCAATCCC \| |
| iNOS | \| F \| CAGCTGGGCTGTACAAACCTT \| \| --- \| --- \| \| R \| CATTGGAAGTGAAGCGTTTCG \| |
| IL-22 | \| F: CATGCAGGAGGTGGTACCTT \| \| --- \| \| R: CAGACGCAAGCATTTCTCAG \| |
| TNF-alfa | \| F: CCCTCACACTCAGATCATCTTCT \| \| --- \| \| R: GCTACGACGTGGGCTACAG \| |
| IL-6 | F: TAGTCCTTCCTACCCCAATTTCC  R: TTGGTCCTTAGCCACTCCTTC |
| GSDMD | \| F \| ATCCTGGCATTCCGAGTGG \| \| --- \| --- \| \| R \| CTCTGGCCCACTGCTTTTCT \| |
| pro-caspase-1 | \| F: CACAGCTCTGGAGATGGTGA \| \| --- \| \| R: CTTTCAAGCTTGGGCACTTC \| |
| ASC | \| F: GACAGTACCAGGCAGTTCGT \| \| --- \| \| R: AGTCCTTGCAGGTCAGGTTC \| |
