# Supplementary material for: Inhibition of GSDMD-mediated pyroptosis triggered by Trichinella spiralis intervention contributes to the alleviation of DSS-induced ulcerative colitis in mice
Source: Parasit Vectors. 2023 Aug 14;16:280. doi: 10.1186/s13071-023-05857-3 (PMC10424392; doi:10.1186/s13071-023-05857-3)
Supplement: Supplementary file 4 — Additional file 4: Table S3. Antibodies used in the western blot experiment. [file 13071_2023_5857_MOESM4_ESM.docx]

**Supplementary table 3**

**Antibodies**

| Antibody | Company and catalog |
| --- | --- |
| NLRP3 | Recombinant Anti-NLRP3 antibody  Abcam (ab263899) |
| IL-1β | Recombinant Anti-IL-1 beta antibody  Abcam (ab254360) |
| GSDMD | Recombinant Anti-GSDMD antibody  Abcam (ab219800) |
| GSDMD-N | GSDMDC1 Antibody (64-Y):  santa cruz sc-81868 |
| Caspase1 | Caspase1 (E2Z1C) Rabbit mAb  CST #24232 |
| Caspase1 p20 | caspase-1 p20 Antibody (D-4)  santa cruz sc-398715 |
| NF-κB (p65) | NF-kB p65 Antibody  SAB Catalog No: #48676 |
| p-NF-κB (p-p65) | Phospho-NF-κB p65 (Ser536) (93H1) Rabbit mAb  CST #3033 |
